# Impact of Microtopography and Neighborhood Effects on Individual Survival Across Life History Stages

**DOI:** 10.3390/plants13223216

**Published:** 2024-11-15

**Authors:** Haonan Zhang, Xiao Zheng, Yi Wu, Baokun Xu, Peng Cui, Xu Zhou, Yanming Fang, Lei Xie, Hui Ding

**Affiliations:** 1 Innovative Research Team for Forest Restoration Mechanisms, Chishui National Ecological Quality Comprehensive Monitoring Stations, Nanjing Institute of Environmental Sciences, Ministry of Ecology and Environment (MEE), Nanjing 210042, China; 2 Research Center for Nature Conservation and Biodiversity, State Environmental Protection Scientific Observation and Research Station for Ecology and Environment of Wuyi Mountains, State Environmental Protection Key Laboratory on Biosafety, Nanjing Institute of Environmental Sciences, Ministry of Ecology and Environment (MEE), Nanjing 210042, China; 3 Co-Innovation Center for Sustainable Forestry in Southern China, College of Life Sciences, Key Laboratory of State Forestry and Grassland Administration on Subtropical Forest Biodiversity Conservation, Nanjing Forestry University, Nanjing 210037, China

**Keywords:** microtopographic variables, neighborhood effects, plant survival, subtropical forest

## Abstract

Understanding drivers of plant community assembly and individual survival in forest ecosystems is crucial for effective conservation and management. While macro-scale factors influencing vegetation patterns are well documented, the combined impact of microtopographic variations and neighborhood effects at neighborhood scales, particularly in subtropical forests, requires further study. To contribute to this area of research, we established a 9.6 ha dynamic plot in a subtropical evergreen broad-leaved forest to examine the interplay between microtopographic factors and neighborhood effects on individual plant survival across different life stages. We conducted a comprehensive analysis of microtopographic variables and neighborhood effects, with individual plant survival censused through repeated surveys at 5-year intervals. Mixed-effects models were employed to assess the combined influence of these factors across life stages. Our results reveal that both microtopographic factors and neighborhood effects significantly influence plant survival, with their impacts varying across life stages. Water availability, represented by flow direction, emerged as a consistently critical factor throughout all life stages. Elevation and the topographic position index showed significant positive effects on survival, particularly in later life stages, possibly reflecting adaptations to light acquisition and water drainage. The influence of topographic factors intensified with succession, while the impact of neighborhood effects, particularly asymmetric competition and conspecific negative density dependence, changed as plants matured. This study enhances our understanding of forest community assembly, emphasizing the importance of considering abiotic and biotic factors across multiple scales for effective forest conservation and management. It provides insights into mechanisms driving spatial variation in community composition, crucial for preserving biodiversity in heterogeneous forest landscapes.

## 1. Introduction

Plants, as autotrophic and sessile organisms, exhibit life-long immobility following establishment, leading to a strong correlation between the spatial distribution of plant species and local environmental factors. At both global and regional scales, climate, topography, and geomorphology play pivotal roles in determining the types and distribution patterns of vegetation [1,2]. Locally, although climatic variables such as precipitation and temperature tend to be more homogeneous, microtopographic variations become pronounced, particularly in tropical and subtropical mountainous forests [3,4,5]. These variations in terrain create diverse microhabitats and microclimates within short distances, significantly influencing vegetation patterns and ecological processes [3,4,5,6,7,8,9]. Investigating such variations is crucial for understanding the drivers of spatial variation in community species composition, especially for the conservation of rare and endangered plants and the management of forests [10,11,12]. Insights from these studies can significantly inform strategies for forest conservation and restoration following environmental disturbances [13].

Topography emerges as a critical factor in shaping forest landscape heterogeneity [3]. Factors like elevation, slope, aspect, and terrain curvature substantially influence environmental conditions such as soil moisture, nutrient availability, sunlight, and microclimate [14,15,16,17]. These factors, in turn, affect plant growth, reproduction, and survival, consequently determining forest structure and composition. Micro-scale topographic variations promote spatial heterogeneity, essential for plant distribution at finer scales [18,19]. Topography acts as a key driver and filter in forest regeneration, influencing species distribution based on specific habitat requirements [20,21,22]. This influence manifests in distinct patterns, with gullies supporting higher biodiversity, ridges favoring drought-tolerant species, and valleys accommodating resource-demanding species [23,24]. Consequently, these topography-induced variations regulate essential resources such as water and light availability, affecting plant survival and growth, which ultimately shapes species distribution and canopy architecture, contributing to the overall forest structure and composition [1,25]. Microtopography can significantly affect the spatial distribution of species by indirectly affecting soil, moisture, and other environmental factors, especially some species with obvious habitat preferences [4,5]. To elucidate the mechanisms underlying these topography-driven patterns, researchers often quantify the influence of environmental regulation on species distribution by examining individual plant survival as explained by environmental variables [4,26,27,28]. This approach provides a crucial pathway for understanding how microtopography impacts forest species distribution patterns, offering insights into the complex interplay between topographic features and plant community dynamics.

Species interactions, such as resource competition and facilitative mutualism, importantly influence the spatial structure of species at neighborhood scales in addition to environmental factors [9,10,11,29,30]. In forests, although the distribution of environmental resources like light, water, and nutrients may appear relatively uniform locally, they are typically limited. Variations in individual plant sizes and local densities lead to differences in competitive abilities. Consequently, smaller plants differ significantly in their resource utilization efficiency compared to larger plants, resulting in a size-proportional (asymmetrical), uneven distribution of these limited resources among individuals [31,32]. This competition is significantly influenced by the density and size of neighboring trees surrounding a focal individual, affecting the resources available for its growth and survival [33,34]. The impact of these neighborhood interactions on the survival–mortality trade-offs in plant individuals is often attributed to density-dependent mortality (NDD), typically categorized into conspecific and heterospecific negative density dependence (CNDD and HNDD) [31,35,36,37,38,39,40]. Furthermore, studies investigating the relationship between biodiversity and individual survival have become more prevalent [41,42,43]. The observed positive correlation between diversity and survival suggests that diversity in heterospecific assemblies at small spatial scales may enhance microclimatic conditions and improve light interception [30,44,45,46].

Collectively, abiotic and biotic factors interact to influence individual survival within forest dynamics, underscoring the importance of considering resource competition and plant interactions in microenvironmental studies. Species interactions exhibit significant scale dependency, with richer interspecies interactions like neighborhood diversity and asymmetrical competition observed at smaller scales. Therefore, analyzing microtopography’s impact on individual survival at the neighborhood level aids in quantifying the synergistic effects of species interactions, as noted by numerous scientists who partition community structure variation into components explained by environmental conditions and species interactions [4,40,47,48,49]. This approach further elucidates the roles of ecological processes such as environmental filtering, competitive exclusion, and mutualistic coexistence in forest dynamics [4,12,28,50,51,52,53]. Quantifying the interactions among forest community structure, individual spatial distribution, environmental factors, and neighborhood effects is thus essential for understanding their driving mechanisms.

To address these challenges, we established a dynamic plot of 9.6 hectares (240 m × 400 m) within a subtropical evergreen broad-leaved forest [54,55], aiming to systematically analyze the effects of microtopography and neighborhood species interactions on tree survival. Based on the preceding literature review and theoretical insights, we propose several scientific hypotheses to guide our research: (H1) Microtopographic factors and neighborhood effects jointly influence the survival of individuals. (H2) The asymmetrical competition resulting from individual size is significantly correlated with plant survival, with larger individuals having a higher probability of survival. (H3) The influence of microtopographic factors and neighborhood effects on survival varies across different life history stages. (H4) Different species exhibit distinct survival probabilities in response to microtopographic conditions and neighborhood effects. Specifically, the 32 dominant species demonstrate significant interspecific differences. This integrated perspective is crucial for advancing conservation and restoration strategies, particularly in heterogeneous forest landscapes.

## 2. Results

### 2.1. Microtopographic and Neighborhood Effects on Tree Survival

**Figure 1:**
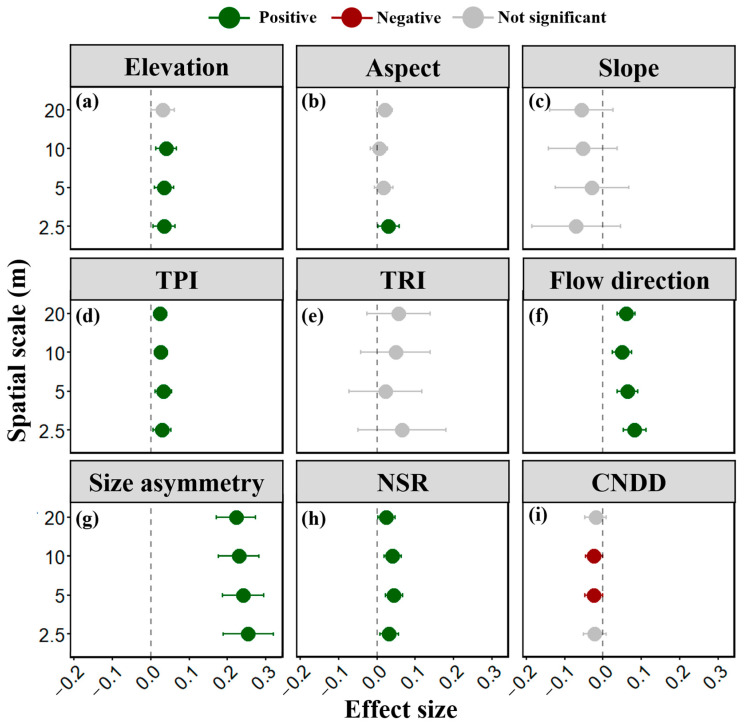
Parameter estimates of microtopographic (**a**–**f**) and neighborhood effects (**g**–**i**) on individual tree survival at neighborhood scales. Dots represent estimated parameter effects, with error bars indicating standard errors. A semi-transparent gray dashed line indicates a null effect (parameter estimate of zero) in each subplot.

Microtopography and neighborhood effects were analyzed for their influence on individual tree survival across different spatial scales. Elevation, topographic position index (TPI), and flow direction consistently exhibited positive effects on survival across all scales, with relationships generally significant (Figure 1a,d,f). This suggests that topographic factors regulating light availability (elevation and TPI) and water availability (flow direction) strongly influence individual survival. Flow direction, in particular, not only significantly promoted survival at all scales but also demonstrated the largest effect size, indirectly indicating that water availability is a crucial determinant of survival. Aspect also positively affected survival probability, but only at smaller scales (Figure 1b). In this study, higher aspect values corresponded to south-facing slopes, while lower values represented north-facing (shaded) slopes. The positive correlation suggests higher survival probabilities for individuals on shaded slopes. While TRI and slope exhibited positive and negative influences, respectively, these effects were not statistically significant (Figure 1c,e).

Regarding neighborhood effects, asymmetric competition based on individual diameter at breast height (DBH) significantly impacted survival. A strong positive correlation was observed between individual DBH and survival rate, indicating higher survival for larger trees (Figure 1g). Conspecific negative density dependence (CNDD) was also detected, demonstrating a significant negative impact on individual survival (Figure 1i). Interestingly, a positive effect of diversity on individual survival was also observed (Figure 1h).

In summary, as hypothesized (H1 and H2), both microtopographic factors and neighborhood effects play a role in influencing individual tree survival. Furthermore, asymmetric competition related to individual size significantly correlates with survival, with larger individuals exhibiting higher survival rates.

### 2.2. Microtopographic and Neighborhood Effects Across Life Stages

**Figure 2:**
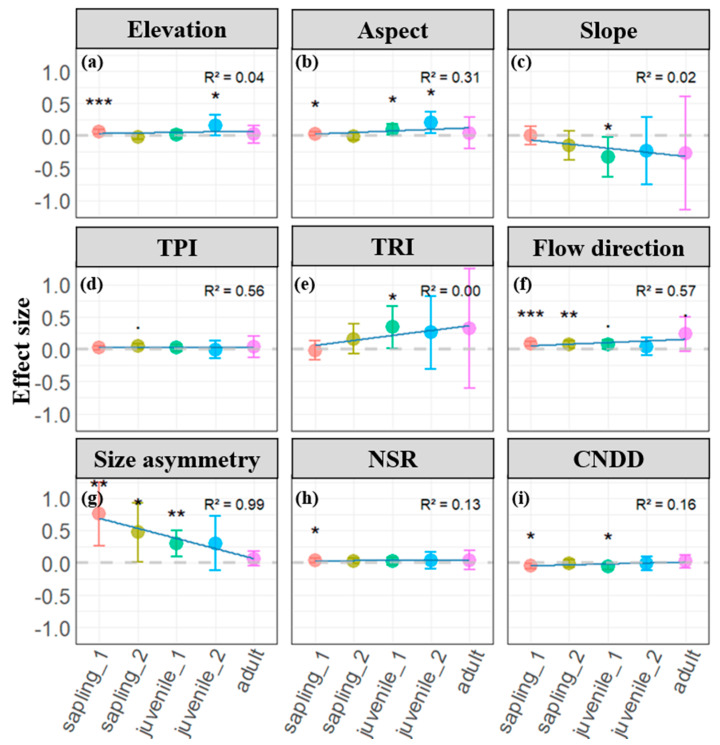
Parameter estimates of microtopographic (**a**–**f**) and neighborhood effects (**g**–**i**) on individual tree survival across different life history stage. Dots represent estimated coefficients with error bars depicting standard errors. Positive coefficients indicate positive effects, while negative coefficients indicate negative effects. Significance levels: • *p* < 0.1; * *p* < 0.05; ** *p* < 0.01; *** *p* < 0.001. In the inset figure, R-squared values represent the regression coefficient for changes across life stages, and light blue lines illustrate the trend of neighborhood effects across life stages.

The influence of microtopography and neighborhood effects, including individual size, on individual tree survival was analyzed across different life history stages. The positive effects of elevation, aspect, TPI, TRI, and flow direction on survival progressively increased from early to late life stages (Figure 2a,b,d–f), while the negative effect of slope intensified with successional stage (Figure 2c). Although some parameter estimates were non-significant at certain stages, this overall trend suggests that as individuals grow and develop, their survival becomes increasingly influenced by these relatively stable environmental factors. Adult trees, in particular, exhibit a stronger dependence on these factors.

In contrast to the shifting influence of environmental factors, the positive effect of asymmetric competition on survival decreased significantly with successional stage and individual growth (Figure 2g). This indicates that asymmetric competition among adult trees has a considerably weaker impact on survival compared to earlier life stages, and the effect is non-significant for adult trees. The analysis of conspecific negative density dependence (CNDD) revealed a significant negative effect on survival, which weakened with life stage progression (Figure 2h). This suggests that adult trees (i.e., larger trees) exhibit greater tolerance to CNDD, although the magnitude of this change is relatively small (R^2^_CNDD_ = 0.13). Neighborhood diversity consistently demonstrated a significant positive effect on survival across life stages, but similarly to CNDD (Figure 2i), the magnitude of change with succession was minimal (R^2^_NSR_ = 0.16).

Overall, these findings support Hypothesis 3, demonstrating that the influence of microtopography and neighborhood effects on survival varies across life history stages. Specifically, the impact of microtopographic factors on individual survival increases from early to late stages, while the influence of neighborhood effects decreases or remains relatively constant.

### 2.3. Interspecific Variability

**Figure 3:**
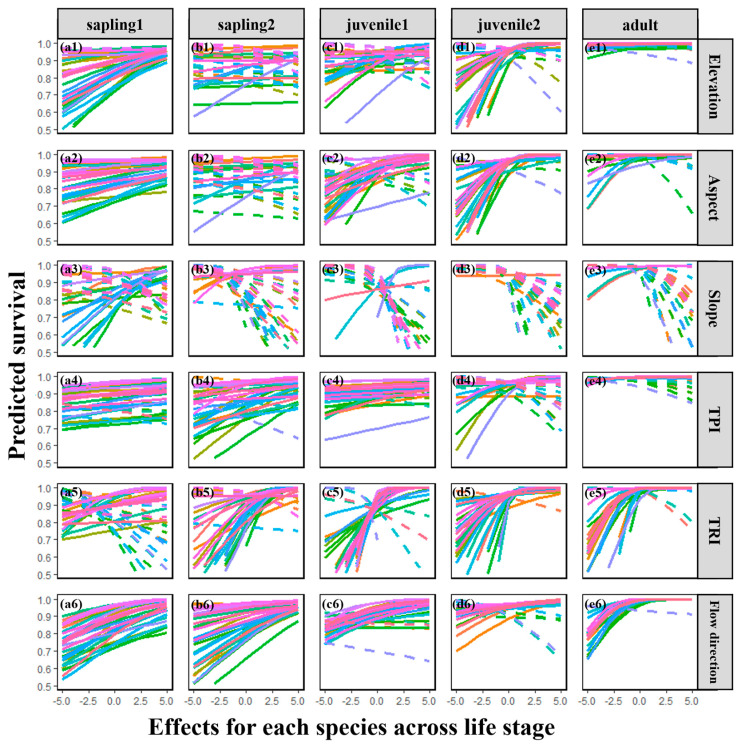
Relationship between six microtopographic factors (elevation: (**a1**–**e1**), aspect: (**a2**–**e2**), slope: (**a3**–**e3**), TPI: (**a4**–**e4**), TRI: (**a5**–**e5**) and flow direction: (**a6**–**e6**)) and survival probability among 32 species across life stages at a scale of 2.5 m. The figure displays predicted survival probability for each of the 32 observed species across life stages at a scale of 2.5 m, and other test scales are 5 m, 10 m, and 20 m; see Figure S3–S5 in the Supplementary Files. Different species are represented by different colors in the lines (see Figure S5 in the Supplementary Files): the solid line represents a positive effect and the dotted line represents a negative effect. Predicted survival probability is back-transformed from the generalized linear mixed model as described in the text, and all neighborhood effects were Z-score-transformed at quantification.

**Figure 4:**
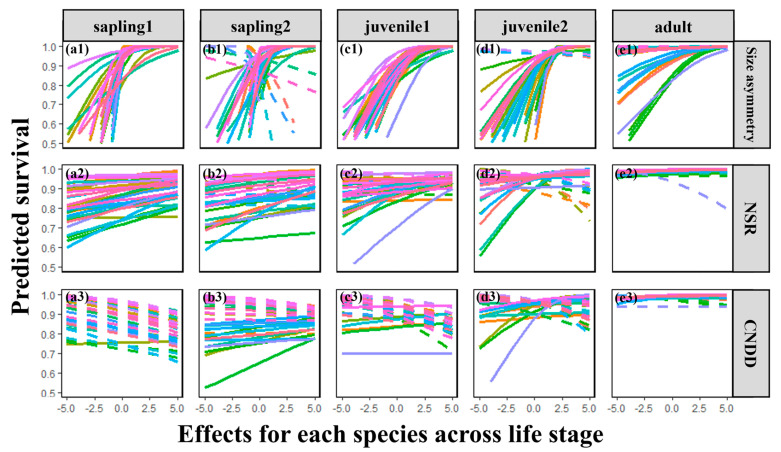
Relationship between three neighborhood effects (size asymmetry: (**a1**–**e1**), NSR: (**a2**–**e2**), and CNDD: (**a3**–**e3**)) and survival probability among 32 species across life stages at a scale of 2.5 m. The figure displays predicted survival probability for each of the 32 observed species across life stages at a scale of 2.5 m, and other test scales are 5 m, 10 m, and 20 m; see Figure S6 in the Supplementary Files. Different species are represented by different colors in the lines (see Figure S3 in the Supplementary Files): the solid line represents a positive effect and the dotted line represents a negative effect. Predicted survival probability is back-transformed from the generalized linear mixed model as described in the text, and all neighborhood effects were Z-score-transformed at quantification.

As hypothesized (H4), different species exhibited varying survival probabilities in response to microtopography and neighborhood effects. Survival probabilities were predicted for 32 dominant tree species using mixed-effects models, revealing significant interspecific variation (Figure 3 and Figure 4). Among microtopographic factors, elevation, aspect, TPI, and flow direction generally exerted positive effects on survival during early life stages, particularly the Sapling_1 stage (Figure 3(a1,a2,a4,a6)). However, this consistency diminished across life stages, suggesting that habitat preferences diverge as individuals grow. Notably, the influence of flow direction remained relatively stable throughout the life cycle, indicating the persistent importance of water availability for survival within the observed timeframe.

Regarding neighborhood effects, the positive influence of asymmetric competition on survival was relatively consistent across species but gradually weakened (decreasing slope) throughout the life cycle. This decline was particularly pronounced in the later adult stage (Figure 4(e1)), which explains the non-significant overall effect observed in Figure 2g. Furthermore, neighborhood species richness (NSR) and CNDD exhibited relatively consistent negative and positive effects, respectively, during the seedling stage (Figure 4(a2,a3)). However, these effects became increasingly variable in later life stages (Figure 4(d2,e2,d3,e3)).

## 3. Discussion

Topography acts as both a driver and filter in forest natural regeneration, significantly influencing species distribution based on specific habitat requirements [20,21,22]. This filtering effect is particularly evident in its impact on individual plant survival during natural regeneration processes [3,4,5]. Characteristically, valleys and lowlands, with their deeper, fertile soils and ample moisture, support species with higher resource demands [3,14,24]. Our study provides empirical evidence for these topographical influences. At scales between 2.5 m and 5 m, individual survival rates were notably higher on shaded aspect (Figure 2b). Furthermore, flow direction significantly promoted individual survival across all tested scales (Figure 2f), supporting the notion that water availability is a crucial factor in individual survival. This finding aligns with previous research indicating that flow direction influences soil moisture distribution, which in turn affects seedling establishment and growth [5]. Areas aligned with optimal flow directions tend to retain more moisture, thereby reducing drought stress and enhancing nutrient uptake efficiency. Consistent water availability can also buffer plants against extreme weather conditions, contributing to higher survival rates [5,56,57]. Interestingly, we also detected significant positive effects of elevation and TPI on individual survival (Figure 2a,d), possibly reflecting the adaptation of light-demanding or drought-tolerant species to ridges with shallower, well-drained soils [15,16,17]. Although we did not directly assess soil characteristics, the existing literature suggests that higher elevations and specific topographic positions often correlate with distinct soil moisture regimes and nutrient availability [56,57,58,59]. For instance, ridges may offer better drainage, which benefits species adapted to lower moisture conditions, while valleys may retain more nutrients and moisture [5]. Future studies incorporating soil analyses would provide a more comprehensive understanding of how elevation and TPI influence plant survival through soil-mediated mechanisms.

Beyond topographical factors, neighborhood effects also significantly influenced individual survival. Notably, DBH showed a strong positive correlation with survival probability, indicating that initial plant size is crucial in determining resource acquisition capabilities within our 5-year monitoring period [5,13,51,52]. Consistent with classic studies, conspecific negative density dependence (CNDD) significantly reduced species survival [31,35,36,37,40]. Conversely, aligning with recent research, diversity promoted individual plant survival [30,44,45,46]. In summary, our study reveals that small-scale topographic factors exhibit strong spatial variation, with their impact on individual survival often rivaling the degree of variation seen across broad environmental or biogeographic gradients [1,2].

The process of plant community assembly is dynamic and unfolds over time, with distinct assembly mechanisms emerging at various stages of plant growth [5,9,27,30,38,39,40,49]. This mechanism is profoundly influenced by external environmental factors and interspecific interactions among individual plants [3,4,5,18,40,49]. Our study quantifies the impact of these influential factors on individual survival across different life history stages, decomposing them into two crucial aspects: microtopography and neighborhood effects. Interestingly, we observed that as succession progresses, plant growth significantly alters the response patterns of individual survival to microtopographic factors and neighborhood effects. Specifically, topographic factors that promote individual survival exhibit stronger positive effects in later life stages, while those with negative impacts show more pronounced inhibitory effects. In particular, the positive effects of elevation, TPI, TRI, flow direction, and aspect on survival intensify with succession, potentially reflecting the increased demand for resources such as light and water as plants grow and their size increases [49]. Conversely, the negative impact of slope on survival strengthens over time, indicating the influence of environmental filtering on individual survival [49]. Notably, despite considerable variation in species’ responses to different topographic factors (Figure 4), flow direction maintains a relatively stable influence throughout the life cycle (Figure 3(a6–e6)). This suggests that water availability remains one of the most critical factors affecting individual survival throughout the observed life history stages.

Furthermore, by examining the effects of asymmetric competition, diversity, and conspecific negative density dependence (CNDD) on individual survival, we observed that plant growth also increases tolerance to competition. We attribute this to varying regulatory mechanisms of asymmetric competition across different life history stages. Initially, we observed a significant positive correlation between individual size and survival probability in early life stages. However, this correlation diminishes as life stages progress, with the promoting effect of individual size on species survival decreasing and becoming non-significant in the mature tree stage (Figure 2g). This indicates the presence of significant asymmetric competition within the plot, where larger individuals possess stronger competitive abilities [5,51,52,53]. As growth and development continue, the disparity in individual sizes gradually decreases, further reinforcing the notion that asymmetric competition among large trees has a reduced impact on individual survival [51]. Notably, the relationships between CNDD, diversity, and survival become less stable in early life stages as life stages progress (Figure 4(d2,e2,d3,e3)). Specifically, plants’ tolerance to CNDD gradually increases with individual growth. This result supports classic conclusions from CNDD research, namely, that negative density-dependent mortality is most pronounced in early life stages of plants [39]. It also aligns with the aforementioned perspective on asymmetric competition, suggesting that competition among mature trees gradually becomes more symmetric, potentially leading to a decrease in density-dependent mortality caused by CNDD [51,53]

While this study provides valuable insights into the effects of topography and neighborhood dynamics on individual plant survival, it is important to acknowledge certain limitations. One notable limitation is the absence of an analysis of soil nutrient availability across the study area. In a landscape covering nearly 10 hectares, soil properties can exhibit significant spatial variability, leading to differences in nutrient availability that may influence plant growth and survival. Soil nutrients play a critical role in sustaining plant health and resilience, and variations in nutrient levels could potentially affect the outcomes observed in our study. Consequently, the omission of soil nutrient analysis may limit the comprehensiveness of our findings. Future research should incorporate detailed assessments of soil nutrient profiles to better elucidate their impact on forest natural regeneration and to provide a more holistic understanding of the factors driving individual plant survival.

## 4. Materials and Methods

### 4.1. Study Area and Plot Establishment

The research was carried out in Wuyishan National Park, situated in the northwest region of Fujian Province, China. This locale experiences an average annual temperature of 19.2 °C and receives roughly 1600 mm of precipitation each year. In this region, red soil is the most extensively distributed zonal soil type, ranging from riverbeds at an elevation of 160 m to mountainous areas up to 1100 m. The predominant vegetation in this area consists of subtropical evergreen broad-leaved forests (Wu, 1980), although past commercial logging has predominantly transformed these original forests into secondary growth [49,54]. The site underwent selective logging of the Chinese fir plantations originally used for timber production, with efforts made to preserve native forest species during the thinning process.

**Figure 5:**
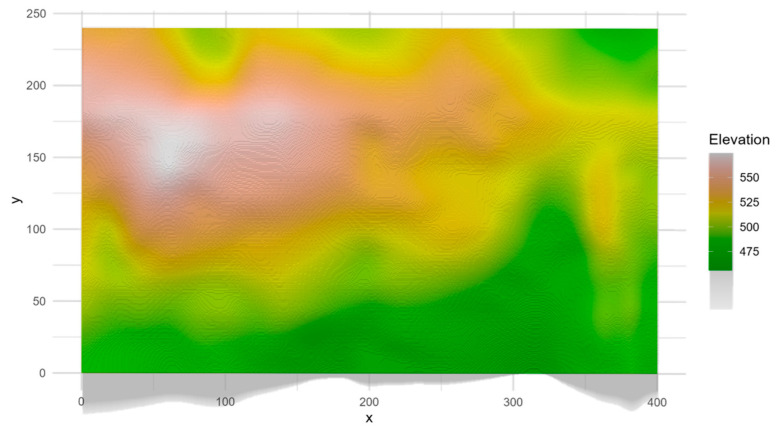
Microtopography in dynamic plot. This map only shows the combined terrain factors and the basic conditions of elevation; specific factors such as aspect and slope are shown in Figure S1 in the Supplementary Files. The shadow effect illustrates terrain undulations in orthographic view.

Within this secondary subtropical evergreen broad-leaved forest, a 9.6-hectare dynamic plot was established in 2013, located at 27°35′24.23′′ N, 117°45′55.43′′ E, and measuring 400 m by 240 m (Figure 5). The plot features moderate topographic variability, with elevations ranging from 450 to 580 m. Formerly part of the Sixin Forestry plantation, the area was subject to logging in the 1960s and has since experienced six decades of natural regeneration.

### 4.2. Repeated Censuses and Individual Tree Survival

In accordance with the CTFS (Center for Tropical Forest Science) survey protocols, the entire plot was divided into 240 large quadrats (20 m × 20 m), and each large quadrat was further subdivided into 16 smaller plots (5 m × 5 m), totaling 3840 small plots. These smaller quadrats were used as work units to measure the relative position, DBH (diameter at breast height), and other individual attributes of all trees. Two censuses were conducted in the dynamic plot between 2013 and 2018. During these censuses, species identity, location, DBH, height, and crown base height were recorded for all trees with DBH ≥ 1 cm. The first census showed a total of 68,336 tree individuals (including branches and sprouts) with DBH ≥ 1 cm, belonging to 173 species, 88 genera, and 48 families. The co-dominant families included Fagaceae, Ericaceae, and Elaeocarpaceae, with co-dominant species including *Castanopsis carlesii*, *Castanopsis fordii*, *Castanopsis eyrei*, and *Schima superba*. No single species was overwhelmingly dominant (Table S1 in the Supplementary Files), and the stand structure indicated that the forest community in our study was still in the early stage of secondary succession because most tree individuals were saplings [49,54]

Tree survival was determined using binary code for each individual (1 for survival, 0 for death), focusing solely on trees alive at the start of the interval to compute survival probability. We selected 32 co-dominant tree species based on their importance values, abundance, and average DBH (diameter at breast height) from the plot for this study (Tables S1 and S2 in the Supplementary Files). Importance values (IVs) were calculated by combining the percentages of relative abundance, relative dominance (DBH), and relative frequency for these 32 co-dominant species, reflecting their overall ecological significance within the community [54,55].

### 4.3. Microtopographic Factors and Neighborhood Effects

Within our dynamic forest plot, fundamental microtopographic variables, including mean elevation, aspect, and slope, were assessed for each subplot (Figure 5 and Figure S1 in the Supplementary Files). Following Center for Tropical Forest Science (CTFS) protocols, the plot was divided into 240 quadrats (20 m × 20 m), each further subdivided into 16 subplots (5 m × 5 m). Elevation, aspect, and slope were measured in each subplot using a total station. These measurements were then used to generate a digital elevation model (DEM) of the plot, from which microtopographic variables were derived [4,49]. Higher aspect values in the DEM correspond to south-facing slopes, while lower values represent north-facing (shaded) slopes. To explore the influence of microtopography on ecological processes within plant communities, we also derived and quantified more complex microtopographic factors from the DEM data, such as the terrain position index (TPI), terrain ruggedness index (TRI), and flow direction [56,57]. The TPI evaluates terrain position by calculating the mean elevation difference between each pixel and all neighboring pixels in a DEM. The TRI quantifies terrain roughness by computing the root mean square of the elevation differences between each pixel and its eight adjacent pixels in a DEM. Flow direction, derived from the DEM, represents the direction of water flow based on elevation differences between a central pixel and its eight neighbors. Higher flow direction values indicate areas of greater water accumulation, suggesting increased water availability for plants in those locations [56,57]. Microtopographic variables for focal tree species at different neighborhood scales were calculated and visualized using R Studio (based on R version 4.2.3), utilizing the packages “spatstat (version 3.0-3)” and “raster (version 3.6-2)”.

Size-asymmetric competition was assessed by examining the relationship between individual tree size (diameter at breast height, DBH) and survival [40,49,51,53]. DBH was measured for each tree, and its location within the plot was recorded (Figure S2a in the Supplementary Files). Repeated censuses over a five-year period tracked individual survival, allowing us to test Hypothesis 2 (H2), which posited that larger individuals (greater DBH) exhibit higher survival rates. DBH was chosen as a proxy for individual size due to its reliability and ease of measurement in large-scale field surveys (with over 60,000 individuals censused per survey). Neighborhood species richness (NS; Figure S2b in the Supplementary Files) assesses biodiversity by counting distinct tree species within a specified radius around each focal tree. For any given focal tree i, NSR is precisely calculated as the total number of immediate heterospecific neighbor species, mathematically represented as ${NSR}_{i}=\sum_{j\neq i}N_{j}$, where N denotes the recorded number of species for each neighboring tree j. This measure allows for an in-depth analysis of how the survival of a focal tree is influenced by the species diversity of its immediate surroundings. The NSR around each individual was calculated for four radii (2.5, 5, 10, or 20 m). The conspecific neighborhood competition indices (CNDD; Figure S2c in the Supplementary Files) were calculated by evaluating the DBH area of neighboring trees of the same species [30,38,39]. The indices were formulated as ${CNDD}_{i}=\sum_{j\neq i}\frac{\pi D_{j}^{2}}{4}$, where Dj represents the diameter at breast height (DBH) of neighboring trees [30,39]. We computed the microtopography factor and neighborhood effect indices for neighborhoods of different radii: 2.5, 5, 10, and 20 m [5,30,39]. Neighborhood effects for focal tree species at different neighborhood scales were calculated and visualized using R Studio (based on R version 4.2.3), utilizing the package “spatstat (version 3.0-3)”.

### 4.4. Microtopographic and Neighborhood Effects on Tree Survival

Generalized linear mixed-effects models (GLMMs) with binomial error structures were used to analyze the influence of microtopographic factors and neighborhood effects on tree survival across life history stages. Microtopographic factors (elevation, slope, aspect, TPI, TRI, and flow direction) and neighborhood effects (size-asymmetric competition, NSR, and CNDD) were included as predictors [5,30,39,40]. To facilitate comparison and improve model stability and interpretability, given the disparate scales of predictors like competition indices and species richness, all predictors were Z-score-transformed. For all individuals, a basic model was fitted at each of life stages, from the focal individual.

$$\begin{array}{ll} \ln\left(p_{ij}/(1-p_{ij})\right) & \\ & ={\;\beta }_{0j}+{{\;\beta }_{1}Elevation\;}_{ijp}+{\;\beta }_{2}{Aspect}_{ijp}+\beta_{3}{Slope}_{ijp}\\ & +\beta_{4}{TPI}_{ijp}+\beta_{5}{TRI}_{ijp}+\beta_{6}{Flow\;direction}_{ijp}\\ & +\beta_{7}{Size\;asymmetric}_{ijp}+{\;\beta }_{8}{NSR}_{ijp}\\ & +\beta_{9}{CNDD}_{ijp}{+\varepsilon }_{j}\;{+p}_{q} \end{array}$$

where $p_{ij}$ is the predicted survival probability of each focal tree i of species j growing in quadrats q. We included all microtopographic factor and neighborhood effect variables as fixed effects in our model. The coefficients ${\;\beta }_{1}$ to ${\;\beta }_{9}$ represent the effects of these variables on survival probability. The random effect structure incorporates crossed random effects for species identity and plot identity to account for the variability in survival probabilities across different species and quadrats (small plots). This includes ${\;\varepsilon }_{j}$, random intercepts and slopes for species *j*. This term accounts for baseline survival probability differences across species and allows the effects of microtopographic factor and neighborhood interaction to vary among species. It also includes ${\;p}_{q}$, random intercepts for quadrats, accounting for potential differences in baseline survival probabilities across different quadrats [30,39,40].

Furthermore, the inclusion of random intercepts and slopes for species *j* in the models allowed us to predict species-specific responses (i.e., survival probabilities) to microtopographic factors and neighborhood interactions. This approach facilitated testing Hypothesis 4, which stated that different species exhibit distinct survival probabilities in response to these factors. We examined the survival responses of 32 dominant tree species to nine predictors. We used the “lme4 1.1-32” package in R to run the basic model for each life stage (based on R version 4.2.3).

## 5. Conclusions

Our study provides crucial insights into the complex dynamics of plant community assembly in forest ecosystems, focusing on the understudied impact of microtopographic variations and neighborhood effects at local scales. We demonstrate that both microtopographic factors and neighborhood effects significantly influence individual plant survival across different life stages in a subtropical evergreen broad-leaved forest. This research reveals that topographic factors, particularly those related to water availability (flow direction) and light acquisition (elevation and TPI), consistently impact survival throughout plant life cycles. Notably, the effects of these factors intensify as succession progresses, reflecting changing resource demands and environmental filtering. At fine scales (2.5 m to 5 m), survival rates were higher on shaded aspects. Elevation and TPI also showed significant positive effects on survival, though the mechanisms behind these relationships require further investigation. We observed that the influence of both topographic factors and neighborhood effects (size-asymmetric competition, CNDD, and NSR) varies across life history stages. As plants mature, their tolerance to competition increases, with the impact of individual size (DBH) on survival probability decreasing in later life stages. Conspecific negative density dependence (CNDD) shows a decreasing influence on mortality in mature plants, supporting classic theories of density-dependent effects being most pronounced in early life stages. These findings underscore the need for multiscale, temporally explicit approaches in forest ecology research and management. By elucidating the interplay between abiotic and biotic factors across plant life stages, our study contributes to a more nuanced understanding of forest community assembly processes. This comprehensive view has important implications for conservation and restoration strategies, particularly in heterogeneous forest landscapes.

One notable limitation of our study is the absence of an analysis of soil nutrient availability across the study area. In a landscape covering nearly 10 hectares, soil properties can exhibit significant spatial variability, leading to differences in nutrient availability that may influence plant growth and survival. We observed that the survival rates of different species are influenced by topographic factors and biotic interactions, reflecting species-specific traits. However, further investigation is needed to understand the underlying causes of these phenomena. Future research could integrate functional trait data to distinguish the resource acquisition strategies of different taxa, such as comparing pioneer species with resource-conservative species in their responses to topographic factors and species interactions. Long-term studies across diverse forest ecosystems are necessary to unravel these complex ecological interactions and inform effective forest management practices in the face of global environmental changes. Understanding these interactions thoroughly will be foundational in formulating strategies that ensure the resilience and sustainability of forest ecosystems.

## Data Availability

The forest census data are available from the corresponding author on reasonable request.

## Supplementary Materials

The following Supplementary Files can be downloaded at: https://www.mdpi.com/article/10.3390/plants13223216/s1, Figure S1. Spatial variation in microtopographic predictors at the neighborhood scale: elevation (a), aspect (b), slope (c), terrain position index (d), terrain ruggedness index (e), and flow direction (f). The maps were generated using an Epanechnikov kernel with a bandwidth of 5, and the intensity values range from blue (low) to purple (high). Figure S2. Spatial variation in neighborhood effect predictors at the neighborhood scale: DBH size asymmetry (a), neighborhood species richness (b), and CNDD (c). The maps were generated using an Epanechnikov kernel with a bandwidth of 5, and the intensity values range from blue (low) to purple (high). Figure S3. Relationship between microtopographic (elevation, aspect, slope, TPI, TRI, and flow direction) and individual survival across life stages at a scale of 5 m. The inter-census predicted survival probability for each of the 32 co-dominant tree species is represented by lines of different colors, with solid lines indicating a positive relationship and dashed lines indicating a negative one. The predicted individual survival was obtained by back-transforming from the general linear mixed models, with all diversity effects quantified by Z-score transformation. Figure S4. Relationship between microtopographic (elevation, aspect, slope, TPI, TRI, and flow direction) and individual survival across life stages at a scale of 10 m. The inter-census predicted survival probability for each of the 32 co-dominant tree species is represented by lines of different colors, with solid lines indicating a positive relationship and dashed lines indicating a negative one. The predicted individual survival was obtained by back-transforming from the general linear mixed models, with all diversity effects quantified by Z-score transformation. Figure S5. Relationship between micro topographic (elevation, aspect, slope, TPI, TRI, and flow direction) and individual survival across life stages at a scale of 20 m. The inter-census predicted survival probability for each of the 32 co-dominant tree species is represented by lines of different colors, with solid lines indicating a positive relationship and dashed lines indicating a negative one. The predicted individual survival was obtained by back-transforming from the general linear mixed models, with all diversity effects quantified by Z-score transformation. Figure S6. Relationship between neighborhood effect (size asymmetry, CNDD, and NSR) and individual survival across life stage at a scale of 5 m, 10 m, and 20m. The inter-census predicted survival probability for each of the 32 co-dominant tree species is represented by lines of different colors, with solid lines indicating a positive relationship and dashed lines indicating a negative one. The predicted individual survival was obtained by back-transforming from the general linear mixed models, with all diversity effects quantified by Z-score transformation. Table S1. Basic characteristics of the 32 co-dominant tree species in a dynamic forest plot in the Wuyi Mountains, China. Note: Species names and family assignments in the table are based on the Flora of China and conform to the accepted names in Plants of the World Online (PoWO; https://powo.science.kew.org/). Table S2. Forest dynamics of the 32 co-dominant tree species in the subtropical evergreen broad-leaved forest plot in the Wuyi Mountains, China, for the years 2013 and 2018.
