# Supplementary material for: Impact of Microtopography and Neighborhood Effects on Individual Survival Across Life History Stages
Source: Plants (Basel). 2024 Nov 15;13(22):3216. doi: 10.3390/plants13223216 (PMC11598057; doi:10.3390/plants13223216)
Supplement: Supplementary file 1 [file plants-13-03216-s001.zip › plants-1007supplementary files.pdf]

Supplementary Files

Figure S1. Spatial variation of micro topographic predictor at the neighborhood scale: elevation (a), aspect (b), slope (c), Terrain Position Index (d), Terrain Ruggedness Index (e), and flow direction (f). The maps were generated using an Epanechnikov kernel with a bandwidth of 5, and the intensity values range from blue (low) to purple (high).

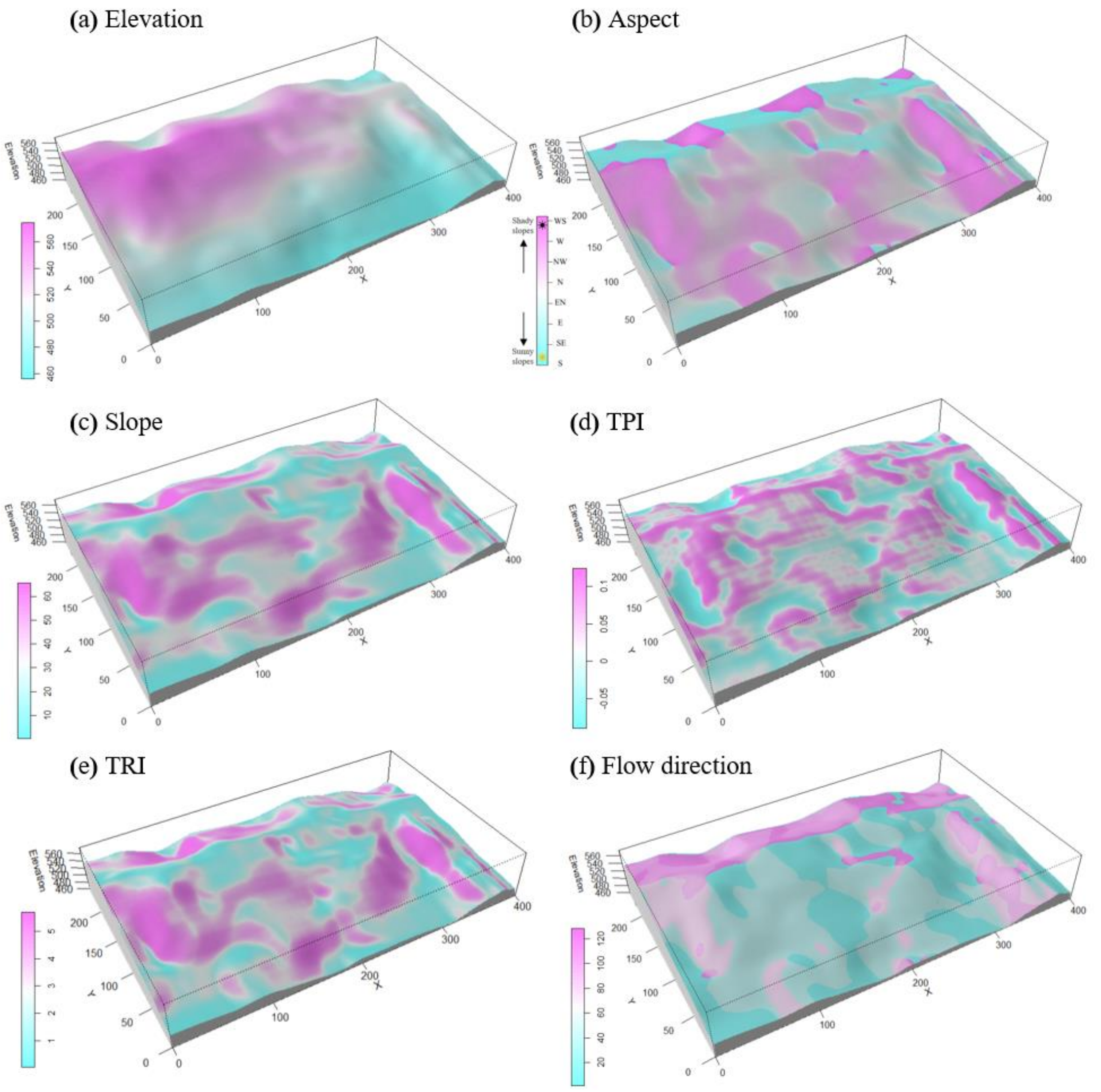

Figure S2. Spatial variation of neighborhood effect predictor at the neighborhood scale: DBH size asymmetry (a), neighborhood species richness (b), and CNDD(c). The maps were generated using an Epanechnikov kernel with a bandwidth of 5, and the intensity values range from blue (low) to purple (high).

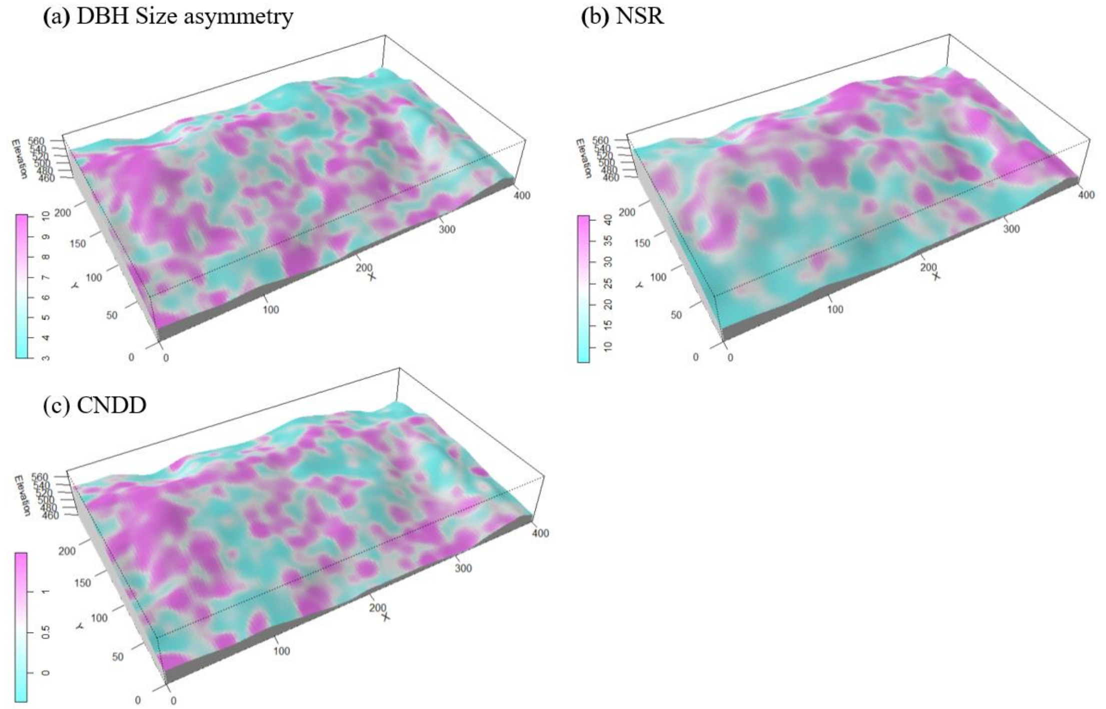

Figure S3. Relationship Between micro topographic (Elevation, Aspect, Slope, TPI, TRI, and Flow direction) and individual survival across life stage at scale of 5 m. It displays inter-census predicted survival probability for each of the 32 co-dominant tree species is represented by lines of different colors, with solid lines indicating a positive relationship and dashed lines indicating a negative one. The predicted individual survival were obtained by back-transforming from the general linear mixed models, with all diversity effects quantified by Z-score transformation.

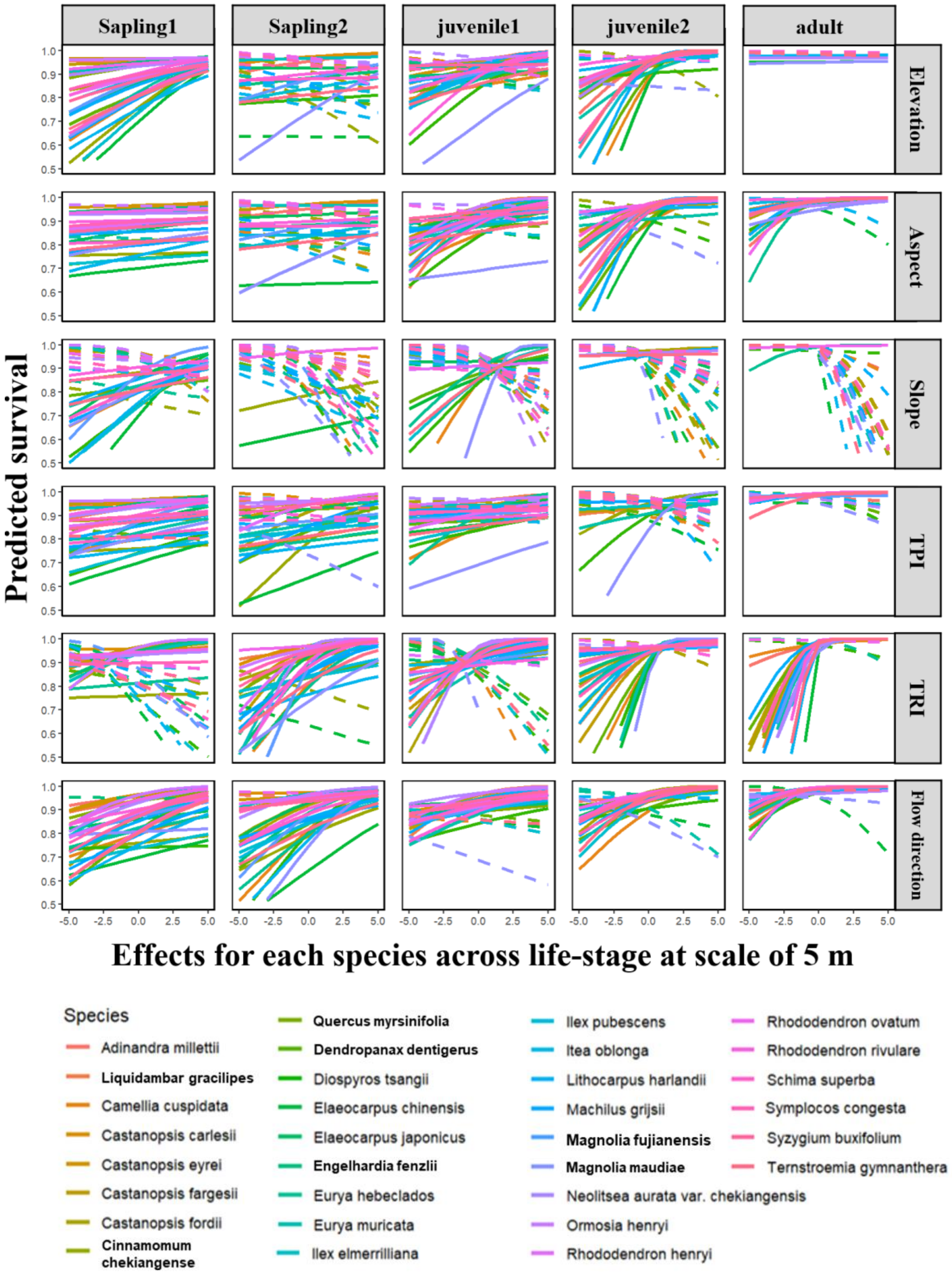

Figure S4. Relationship Between micro topographic (Elevation, Aspect, Slope, TPI, TRI, and Flow direction) and individual survival across life stage at scale of 10 m. It displays inter-census predicted survival probability for each of the 32 co-dominant tree species is represented by lines of different colors, with solid lines indicating a positive relationship and dashed lines indicating a negative one. The predicted individual survival were obtained by back-transforming from the general linear mixed models, with all diversity effects quantified by Z-score transformation.

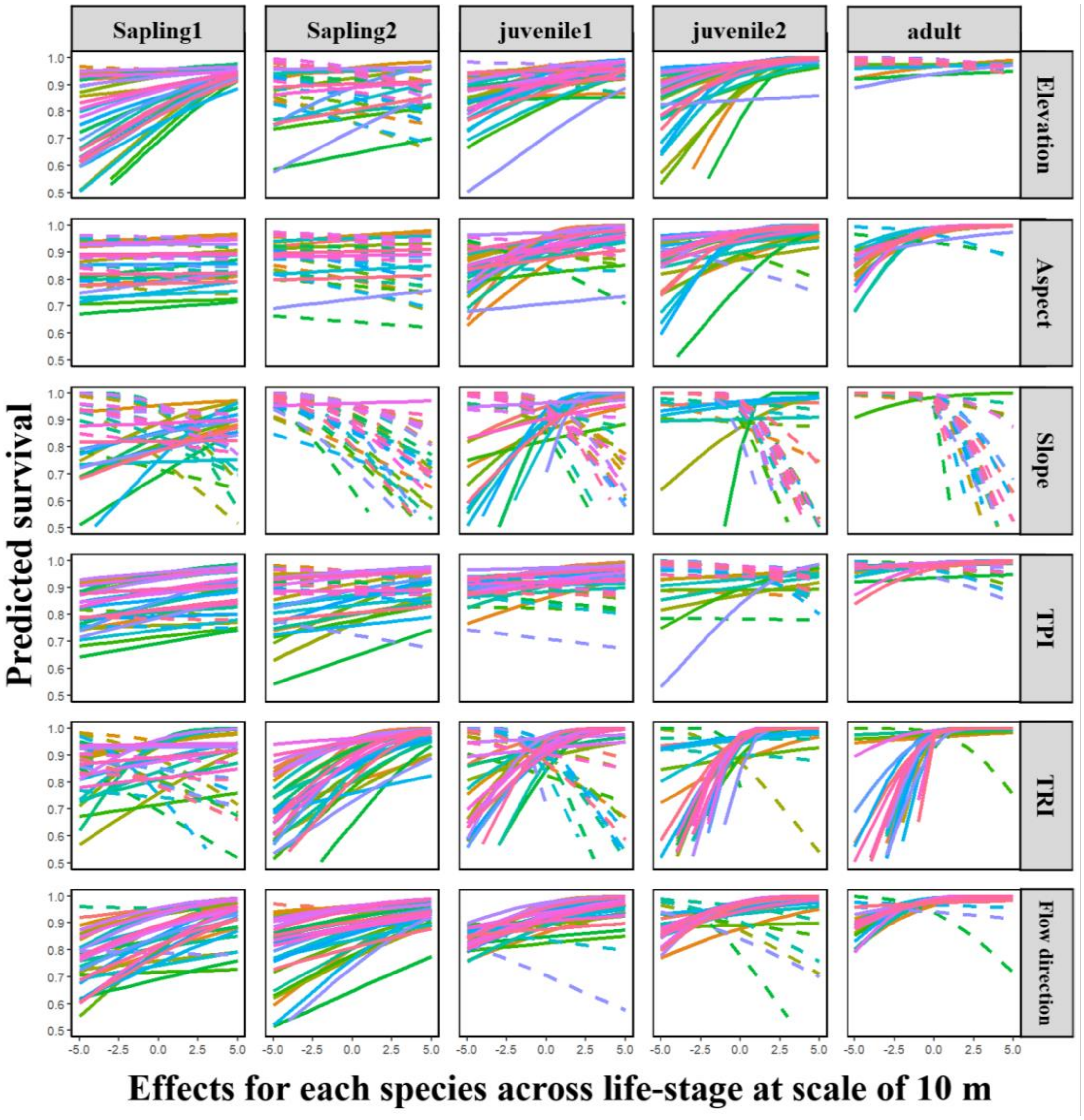

Figure S5. Relationship Between micro topographic (Elevation, Aspect, Slope, TPI, TRI, and Flow direction) and individual survival across life stage at scale of 20 m. It displays inter-census predicted survival probability for each of the 32 co-dominant tree species is represented by lines of different colors, with solid lines indicating a positive relationship and dashed lines indicating a negative one. The predicted individual survival were obtained by back-transforming from the general linear mixed models, with all diversity effects quantified by Z-score transformation.

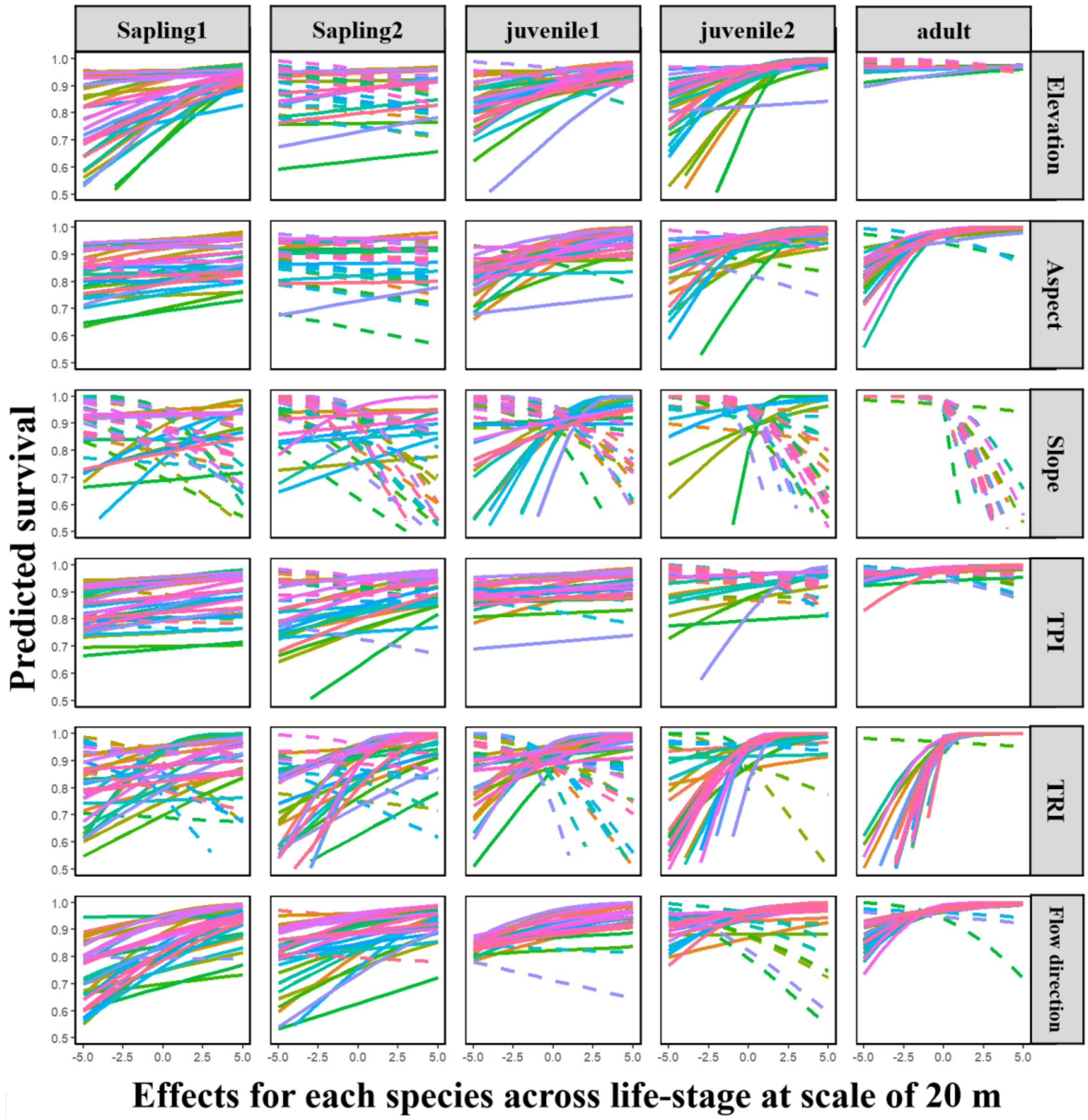

Figure S6. Relationship Between neighborhood effect (Size asymmetry CNDD, NSR) and individual survival across life stage at scale of 5 m, 10 m, and 20m. It displays inter-census predicted survival probability for each of the 32 co-dominant tree species is represented by lines of different colors, with solid lines indicating a positive relationship and dashed lines indicating a negative one. The predicted individual survival were obtained by back-transforming from the general linear mixed models, with all diversity effects quantified by Z-score transformation.

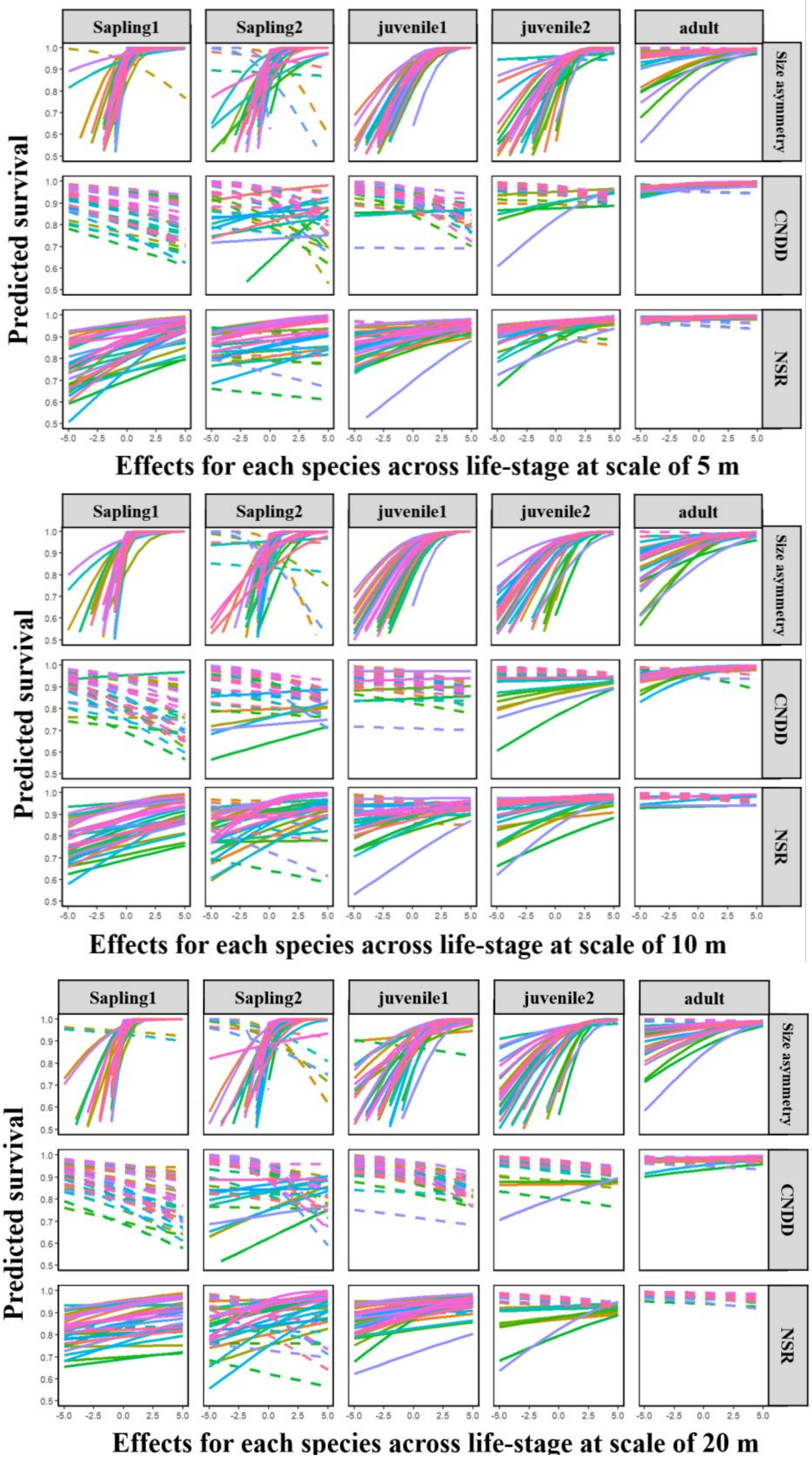

Table S1. Basic characteristics of the 32 co-dominant tree species in dynamic forest plot in the Wuyi Mountains, China. Note: Species names and family assignments in the table are based on the Flora of China and conform to the accepted names in Plants of the World Online (PoWO; https://powo.science.kew.org/).

Species	family	Importance Value (%)	Average of DBH (cm)
Castanopsis carlesii	Fagaceae	7.16	11.65 ± 0.26
Castanopsis fordii	Fagaceae	5.29	7.52 ± 0.13
Castanopsis eyrei	Fagaceae	4.57	11.44 ± 0.27
Engelhardia fenzlii	Juglandaceae	4.37	7.57 ± 0.14
Syzygium buxifolium	Myrtaceae	3.67	2.89 ± 0.05
Schima superba	Theaceae	3.16	7.96 ± 0.20
Rhododendron henryi	Ericaceae	2.91	3.52 ± 0.05
Itea omeiensis	Iteaceae	2.89	2.83 ± 0.03
Eurya muricata	Theaceae	2.78	2.53 ± 0.04
Liquidambar gracilipes	Hamamelidaceae	2.66	6.17 ± 0.18
Magnolia fujianensis	Magnoliaceae	2.49	3.72 ± 0.06
Elaeocarpus japonicus	Elaeocarpaceae	2.44	13.03 ± 0.26
Camellia cuspidata	Theaceae	2.33	2.06 ± 0.02
Castanopsis fargesii	Fagaceae	2.25	11.57 ± 0.40
Elaeocarpus chinensis	Elaeocarpaceae	2.12	8.58 ± 0.20
Lithocarpus harlandii	Fagaceae	2	7.19 ± 0.18
Ormosia henryi	Fabaceae	1.75	3.82 ± 0.09
Machilus grijsii	Lauraceae	1.65	2.02 ± 0.04
Eurya hebeclados	Theaceae	1.55	2.49 ± 0.03
Symplocos congesta	Symplocaceae	1.43	3.56 ± 0.09
Quercus myrsinifolia	Fagaceae	1.36	6.84 ± 0.37
Magnolia maudiae	Magnoliaceae	1.35	4.34 ± 0.14
Dendropanax dentigerus	Araliaceae	1.31	6.33 ± 0.17
Rhododendron ovatum	Ericaceae	1.2	3.84 ± 0.07
Cinnamomum chekiangense	Lauraceae	1.2	3.52 ± 0.12
Adinandra millettii	Theaceae	1.09	3.74 ± 0.12
Ilex elmerrilliana	Aquifoliaceae	1.02	3.65 ± 0.10
Ilex pubescens	Aquifoliaceae	1.01	1.94 ± 0.04
Ternstroemia gymnanthera	Theaceae	0.94	3.28 ± 0.07
Diospyros tsangii	Ebenaceae	0.91	3.14 ± 0.11
Neolitsea aurata var. chekiangensis	Lauraceae	0.87	3.39 ± 0.09
Rhododendron rivulare	Ericaceae	0.81	2.31 ± 0.03

Table S2. Forest Dynamic of the 32 co-dominant tree species in the Subtropical Evergreen Broad-Leaved Forest plot in the Wuyi Mountains, China for the Years 2013 and 2018

Year	Number Of live trees	Species richness	Mortality rate (%)
2013	50765	32	10.83
2018	45266	32	
